# Utilization of Doxycycline Postexposure Prophylaxis at a Midwestern United States HIV/PrEP Clinic

**DOI:** 10.1093/ofid/ofaf062

**Published:** 2025-01-30

**Authors:** Shawnalyn W Sunagawa, Gabriel Codling, Elizabeth Lyden, Sara H Bares, Kimberly K Scarsi, Joshua P Havens

**Affiliations:** College of Pharmacy, University of Nebraska Medical Center, Omaha, Nebraska, USA; College of Pharmacy, University of Nebraska Medical Center, Omaha, Nebraska, USA; College of Public Health, University of Nebraska Medical Center, Omaha, Nebraska, USA; Division of Infectious Diseases, University of Nebraska Medical Center, Omaha, Nebraska, USA; College of Pharmacy, University of Nebraska Medical Center, Omaha, Nebraska, USA; Division of Infectious Diseases, University of Nebraska Medical Center, Omaha, Nebraska, USA; College of Pharmacy, University of Nebraska Medical Center, Omaha, Nebraska, USA; Division of Infectious Diseases, University of Nebraska Medical Center, Omaha, Nebraska, USA

**Keywords:** doxycycline, *Neisseria gonorrhoeae*, *Chlamydia trachomatis*, *Treponema pallidum*, postexposure prophylaxis, sexually transmitted infections

## Abstract

Appropriate, protocol-adherent, doxycycline postexposure prophylaxis prescribing occurred for 70% of prescriptions from our clinic. Most of the nonadherent prescribing was due to missed sexually transmitted infection screenings (89%). As utilization of doxycycline postexposure prophylaxis continues to increase, it is necessary to ensure appropriate follow-up and monitoring.

## Teaser

This retrospective study on doxycycline post-exposure prophylaxis (doxy PEP) adherence underscores the need for comprehensive sexually transmitted infection screening and monitoring with each prescription. As doxy PEP utilization expands, it is necessary to ensure appropriate patient follow-up.

## BACKGROUND

Doxycycline postexposure prophylaxis (doxy PEP) has been demonstrated to decrease the incidence of sexually transmitted infections (STIs) caused by *Neisseria gonorrhoeae* (GC), *Chlamydia trachomatis* (CT), and *Treponema pallidum* (syphilis), prompting the Centers for Disease Control and Prevention (CDC) to publish clinical guidelines for the use of doxy PEP in June 2024 [1–4]. Key recommendations in the CDC's doxy PEP guidelines include the need for follow-up every 3–6 months to reassess continued doxy PEP utilization, screening for STIs every 3–6 months (including syphilis and GC/CT at all anatomic sites of exposure), prescribing enough doses of doxy PEP based on individual patient and provider shared-decision assessment, and HIV screening in individuals without HIV according to current guideline recommendations. Limited real-world data support the clinical effectiveness of doxy PEP [5, 6] and little is known about doxy PEP prescribing patterns, especially in relation to these key CDC recommendations. To address this, in early 2023, our multidisciplinary team (physicians, advanced practice providers, pharmacists, and nurses) at the University of Nebraska Medical Center's Specialty Care Center (UNMC SCC) developed a doxy PEP protocol and provided targeted education to the clinic providers on appropriate prescribing and screening. Thus, we aimed to evaluate appropriate doxy PEP prescribing and monitoring following implementation of a doxy PEP protocol at the UNMC SCC, an academic ambulatory clinic that provides both HIV care and HIV preexposure prophylaxis (PrEP) care.

## METHODS

As a quality improvement project, per SQUIRE guidelines [7], we retrospectively evaluated patients receiving doxy PEP prescriptions between 1 March 2023 and 29 February 2024. Patients receiving doxycycline for other indications were excluded. The primary outcome was adherence to the UNMC doxy PEP protocol, which closely followed current CDC recommendations [1] (Supplementary material). Adherence to the protocol was defined as STI screenings every 3–6 months, including anatomic site GC/CT and syphilis, or documentation justifying deferral of STI screening; guideline-directed HIV screening for individuals without HIV [8]; or viral load monitoring for individuals with HIV [9]. Secondary outcomes included total doxy PEP doses prescribed, proportion of days covered [10], STI incidence in the doxy PEP cohort compared to background STI incidence in the UNMC SCC during 2023, and factors associated with protocol adherence or incident STIs while prescribed doxy PEP. Proportion of days covered was calculated as the total number of doses prescribed to the patient during the study period divided by the total number of days in the study period for each patient based on their initial prescription date. The background UNMC SCC STI incidence was determined based on total number of positive STI screenings (GC, CT, and syphilis) divided by total number of STI screenings ordered and excluding those in the doxy PEP cohort. We performed descriptive statistics for cohort characteristics and outcomes. Fisher exact test or Student *t*-test were used to assess associations of patient characteristics with protocol adherence or incident STIs. All analyses were completed using SAS, version 9.4 (SAS Institute Inc.) and *P* values <.05 were considered statistically significant. Our institutional review board deemed this project exempt from review.

## RESULTS

There were 90 patients who received a doxy PEP prescription during our study period. Most were male (97.8%) with an average age of 38.6 years. Approximately half of our patients were seen for HIV PrEP care and the other half were seen for HIV-associated care. Patients had a median of 1 documented STI (range 0–6) in the 12 months before initial doxy PEP prescription. Additional cohort characteristics are listed in Table 1. Of the 90 patients receiving doxy PEP prescriptions, 30% were not prescribed in adherence with the UNMC SCC doxy PEP protocol (Table 2). The most common reason was missed STI screenings (89%) and syphilis was most frequently missed. The median doses prescribed on the initial doxy PEP prescription was 5 (range 1–30) with 2 refills being the most common (35.6%) on the initial prescription. Median proportion of days covered was 32% (range 2%–100%). There were 145 GC/CT and 163 syphilis screenings obtained from 90 patients with 13% of patients on doxy PEP diagnosed with an STI. Compared to the background incidence rate, there was no significant difference in the incidence of GC/CT (*P* > .05), but patients prescribed doxy PEP had significantly lower incidence of syphilis (*P* = .01) (Table 2). The only characteristic associated with doxy PEP protocol adherence was a higher number of doses prescribed in the initial doxy PEP prescription (*P* = .004; Supplementary Table 1). Younger mean age (39.6 vs 32.2 years; *P* = .022), reason for care (HIV-associated = 76.9% vs HIV PrEP = 23.1%; *P* = .038), and number of STIs within 12 months of first doxy PEP prescription (0 STI = 7.7%, 1 STI = 46.2%, >1 STI = 46.2%; *P* = .019) were significantly associated with an STI diagnosis while on doxy PEP (Supplementary Table 1).

**Table 1.** Doxy PEP Cohort Characteristics

	N = 90
Gender, no. (%)	
Male	88 (97.8%)
Transgender female	2 (2.2%)
Age (y), mean ± SD	38.6 ± 10.4
Social Deprivation Index, median (range)	44 (1–98)
Race, no. (%)	
White	62 (68.9%)
Black	14 (15.6%)
Asian	3 (3.3%)
Multiracial	2 (2.2%)
Other	9 (10%)
Ethnicity, no. (%)	
Non-Hispanic	72 (80%)
Hispanic	18 (20%)
Insurance coverage, no. (%)	
Commercial/private	57 (63.3%)
Government-funded/ADAP	26 (28.9%)
Uninsured	7 (7.8%)
Reason for care at UNMC SCC, no. (%)	
PrEP	46 (51.1%)
HIV care	44 (48.9%)
Number of STIs within 12 mo prior to initial doxy PEP Rx, median (range)	1 (0–6)
Patient initiated request for Rx, no. (%)	36 (40%)
Provider ordering Rx, no. (%)	
Pharmacist	47 (52.2%)
APP	28 (31.1%)
ID Fellow	13 (14.4%)
ID Attending	2 (2.2%)

Abbreviations: ADAP, AIDS Drug Assistance Program; APP, advanced practice provider; doxy PEP, doxycycline postexposure prophylaxis; ID, infectious diseases; PrEP, preexposure prophylaxis; Rx, prescription; UNMC SCC, University of Nebraska Medical Center Specialty Care Center; SD, standard deviation; STI, sexually transmitted infections.

**Table 2.** Primary and Secondary Outcomes

Primary Outcome (N = 90)	
Doxy PEP Rx in adherence with UNMC protocol, no. (%)	63 (70%)
Missed STI screenings, no.	24
Syphilis	16
Gonorrhea/Chlamydia	3
Both (Gonorrhea/Chlamydia and Syphilis)	5
Non-guideline directed HIV screenings or viral load monitoring, no.	3

Secondary Outcomes (N = 90)	
Participant with incident STI while on doxy PEP, no. (%)	12 (13.3%)
Gonorrhea infection, no.	6
Chlamydia infection, no.	3
Syphilis infection, no.	2
Multiple STIs, no.	1
Proportion of days covered (%), median (range)	32 (2%–100%)
Doses prescribed on initial doxy PEP Rx, median (range)	5 (1–30)
Refills prescribed on initial doxy PEP Rx, no. (%)	
0	27 (30%)
1	27 (30%)
2	32 (35.6%)
3	4 (4.4%)

STI incidence^a^, no. (%)			
	doxy PEP	UNMC	*P*-value
Gonorrhea	7/145 (4.8%)	68/1061 (6.4%)	.58
Chlamydia	4/145 (2.8%)	76/1061 (7.2%)	.13
Syphilis	3/163 (1.8%)	95/1375 (6.9%)	.01

Abbreviations: doxy PEP, doxycycline post-exposure prophylaxis; no., number; Rx, prescription; STI, sexually transmitted infections.

^a^STI incidence defined as positive STI test divided by total number of STI tests obtained; UNMC incidence reflective of 2023 clinic rates.

## DISCUSSION

To our knowledge, this is the first study assessing doxy PEP prescribing and utilization patterns, both of which are emphasized in the CDC doxy PEP clinical guidelines [1, 11]. Appropriate doxy PEP prescribing patterns occurred for 70% of participants in our cohort. These data demonstrate a positive early indicator for uptake of the CDC's 2024 doxy PEP clinical guidelines [1]. However, prescriptions with missed STI screenings were the most common reason for protocol nonadherence. Although this was emphasized during protocol education, we hypothesize that this was partly due to concern for patient-associated cost for STI screenings. Although only 8% of patients were completely uninsured, at our institution, STI screenings can still be cost prohibitive for patients even with insurance coverage. Currently, our clinic has a partnership with our county health department, which provides free GC/CT screenings but are unable to offer free syphilis screenings. These findings emphasize the importance of finding affordable routine STI screening options to ensure the continued effective and appropriate use of doxy PEP. Furthermore, consistent and routine STI screenings are critical for timely STI diagnosis and treatment. A growing concern with wider spread doxy PEP implementation and adoption is the risk of its contribution to the development of antimicrobial resistance [11, 12]. The clinical trials that supported the utilization of doxy PEP for STI prevention tracked antimicrobial resistance; however, this was for a limited follow-up period [2–4]. As utilization of doxy PEP expands, it will be necessary to monitor for potential trends in antimicrobial resistance, which can only be achieved, in part, if patients are being routinely screened and monitored for STIs.

Our cohort demonstrated a significant decrease in incident syphilis infections compared to the previous year's UNMC SCC STI rate. We note the missed opportunity to screen for syphilis in most (21 of 24; 87.5%) nonadherent protocol prescriptions, which may also have been reflective of our clinic's overall syphilis screenings practices. However, this significant reduction in syphilis remains consistent with both the clinical trial and real-world implementation of doxy PEP [2–6]. Additionally, this was in spite of very high rates of syphilis in the state of Nebraska, where syphilis incidence has increased by 364% over recent years and continues to rise [13]. Doxy PEP, when prescribed alongside routine STI screenings and consistent patient follow-up, is an important tool to combat the rise in syphilis. Although our cohort did not demonstrate a significant decrease in new GC/CT infections, this could be attributed to our small cohort size and limited follow-up duration. Younger age, patients receiving care for HIV, and being diagnosed with ≥1 STI in the past 12 months were also associated with being more likely to be diagnosed with an STI while on doxy PEP. Although these factors were statistically significant, we believe it is more important and necessary that doxy PEP be prescribed in the context of comprehensive sexual health education and shared decision making with all patients considering utilizing doxy PEP.

Finally, we observed surprising variability in the number of doxy PEP doses prescribed on the initial prescription and proportion of days covered. The median number of doses prescribed was 5 per prescription with 2 refills, and prescriptions (including refills) were typically covered for a median 32% of days in the study period. Although frequency of utilization in our cohort aligned with the median usage from clinical trials (median 4–7 doses/month), real-world utilization has shown that some patients have more frequent anticipated use (ie, daily dosing) [2, 3, 11]. This individualized, patient-specific prescribing based on behavioral assessment highlights the importance of the CDC's recommendation to provide individualized prescription quantity to last until the patient's follow-up visit. We are mindful that this individualized dose/refill prescribing and lack of prescription standardization may pose challenges regarding refill requests before the patient's next follow-up visit and standardized 30-day supplies of doxy PEP could also improve medication access by removing these previously addressed barriers. However, we believe behavioral/individualized conversations can provide further insight and can help structure and inform follow-up visit discussions surrounding anticipated utilization and updated doxy PEP prescriptions.

This was a retrospective, single-institution quality improvement study with a relatively short duration of follow-up. These limitations may have minimized our ability to capture the full impact of doxy PEP prescribing on long-term STI prevention. However, early assessment of adherence to our clinic's doxy PEP protocol allowed us to re-educate and intervene on inappropriate prescribing prior to increased utilization. We emphasized provider and patient education on the importance of follow-up and routine STI screening to better align with both our clinic protocol and the CDC's doxy PEP clinical guidelines [1].

Our study emphasizes both the early success as well as potential protocol/guideline adherence challenges with doxy PEP utilization in a clinic-based setting. Missed STI screenings remain a critical gap in care that could potentially undermine both doxy PEP's long-term efficacy as well as potential increased risk of antimicrobial resistance. As utilization expands, ongoing education, engagement, and adherence to clinic and/or CDC guidelines for regular follow-up and monitoring is essential in supporting doxy PEP for prevention of STIs.

## Supplementary Material

ofaf062_Supplementary_Data

## Supplementary Data

Supplementary materials are available at *Open Forum Infectious Diseases* online. Consisting of data provided by the authors to benefit the reader, the posted materials are not copyedited and are the sole responsibility of the authors, so questions or comments should be addressed to the corresponding author.

## Notes

***Author contributions.*** S.S., G.C., S.B., K.S., and J.H. contributed to conception and methodology of the project. S.S. and G.C. organized the database. J.H. validated the data. E.L. performed the formal analysis. S.S. wrote the first draft of the manuscript. S.B., K.S., and J.H. oversaw the project. All authors contributed to the reviewing and editing of the manuscript and approved the submitted version.

***Patient Consent Statement.*** Per the University of Nebraska Medical Center institutional review board, this project does not include factors necessitating patient consent.

***Financial support.*** No financial support was provided relevant to this article.
