# Supplementary material for: Utilization of Doxycycline Postexposure Prophylaxis at a Midwestern United States HIV/PrEP Clinic
Source: Open Forum Infect Dis. 2025 Jan 30;12(2):ofaf062. doi: 10.1093/ofid/ofaf062 (PMC11832039; doi:10.1093/ofid/ofaf062)
Supplement: ofaf062_Supplementary_Data [file ofaf062_supplementary_data.docx]

**Utilization of Doxycycline Post-Exposure Prophylaxis at a Midwestern United States HIV/PrEP Clinic**

**Supplemental Materials**

**Supplemental Material**

**SCC doxy PEP Protocol for STI Prevention**

Summary:

In the DoxyVac and DoxyPEP trial, use of doxycycline as a single dose after condomless sexual activity among men who have sex with men (MSM) and transgender women (TGW) significantly reduced the incidence of chlamydia (CT), gonorrhea (GC), and syphilis.

Indications for doxy PEP:

1. Use a shared decision-making approach to for doxy PEP initiation for the following populations:
   1. Men who have sex with men (MSM) and transgender women (TGW) who have had ≥1 bacterial STI in the past 12 months *and/or* are at increased risk for bacterial STIs *and/or* request doxy PEP.
2. Provide comprehensive preventative sexual health counseling and education to all sexually active individuals to include HIV/STI screening, doxy PEP, HIV pre-exposure prophylaxis (PrEP), HIV post-exposure prophylaxis (PEP), vaccinations (e.g., Hepatitis A/B, HPV, Mpox, MenACWY), expedited partner therapy, and/or contraception where warranted.

Evidence:

1. DoxyVac (France)/DoxyPEP (US): Randomized controlled trial using a single, oral dose of doxycycline 200mg within 72 hours after condomless oral, anal, or vaginal sex in MSM and TGW, who were either persons living with HIV (PLWH) or taking HIV PrEP, showed significant reductions in CT (74-89%), GC (51-57%%), and syphilis (77-87%) compared to placebo.
2. dPEP: doxy PEP among 449 cisgender women in a Kenyan study showed no significant reductions in STI incidence despite pharmacokinetic data suggesting sufficient doxycycline concentrations in vaginal fluid.
3. Further studies – including assessments of adherence – are needed to better understand the reasons why doxy PEP was found to be ineffective among women in the Kenyan study. Use of doxy PEP in transgender men has not been studied.

Safety:

Taking doxycycline is well tolerated with low rates of adverse events in the DoxyVac trial and other trials of doxycycline use for other indications (e.g., acne treatment or malaria prophylaxis).

Concerns:

1. Resistance: GC (e.g., reservoir for tetracycline resistant plasmids) and *Staphylococcus aureus* (MRSA). There are unknown effects of doxy PEP on the gut microbiome.

Prescription:

Prescribing doxy PEP: Doxycycline is not FDA-approved for STI PEP and formal national organizational guidance for its use as STI prevention is pending. However, Centers for Disease Control and Prevention (CDC) has released considerations for doxy PEP as an STI preventative strategy.

1. Prescribe 200 mg of doxycycline (#2 100mg capsules) to be taken as soon as possible (up to 72 hours) after condomless oral, anal, vaginal, or neovaginal sex that might lead to STI acquisition. No more than 200 mg every 24 hours.
2. Prescription quantity max of 30 doses per month (#60 100mg capsules or #30 200mg capsules).
   1. Note: 100mg capsules are less costly than 200mg capsules; 200mg capsules are not readily stocked at pharmacies.
3. Avoid vitamins/supplements containing positive cations (e.g., calcium) within 2 hours of taking doxycycline.
4. Consider hematopoietic, renal, and hepatic laboratory monitoring as clinically indicated in addition to counseling patients on standard precautions and warnings while taking doxy PEP, as outlined in the drug package insert (e.g., sun sensitivity, pill esophagitis, and, rarely, intracranial hypertension; refractory headaches or vision changes should be evaluated by a provider).
5. For clinical pharmacists, doxy PEP orders may be prescribed in concordance with the UNMC Specialty Care Center Collaborative Practice Agreement

Screening:

1. Screen for GC and CT at all anatomic sites of exposure (urogenital, pharyngeal, and/or rectal), syphilis, and HIV (if not known PWH) at initiation of doxy PEP and every 3-6 months while on doxy PEP or as indicated.
   1. For PWH, viral load monitoring per guidelines or as indicated
2. Screen for HCV annually.
3. If diagnosed with an STI, treat according to CDC STI treatment guidelines.

**Supplemental Table 1:** Univariable Analysis

|  | **Prescription Not in Concordance with Protocol**  **(N=27)** | **Prescription in Concordance with Protocol**  **(N=63)** | **P-value** |
| --- | --- | --- | --- |
| **Age (years), mean + SD** | 38.6 + 9.4 | 38.7 + 10.8 | 0.961 |
| **Race, no. (%)** |  |  | 1 |
| White | 19 (70.4%) | 43 (68.3%) |  |
| Black | 4 (14.8%) | 10 (15.9%) |  |
| Other | 4 (14.8%) | 10 (15.9%) |  |
| **Ethnicity, no. (%)** |  |  | 1 |
| Non-Hispanic | 22 (81.5%) | 50 (79.4%) |  |
| Hispanic | 5 (18.5%) | 13 (20.6%) |  |
| **Insurance coverage, no. (%)** |  |  | 0.351 |
| Commercial/Private | 19 (70.4%) | 38 (60.3%) |  |
| Government-funded/ADAP | 5 (18.5%) | 21 (33.3%) |  |
| Uninsured | 3 (11.1%) | 4 (6.3%) |  |
| **Reason for care at SCC, no. (%)** |  |  | 0.067 |
| PrEP | 18 (66.7%) | 28 (44.4%) |  |
| HIV | 9 (33.3%) | 35 (55.6%) |  |
| **Number of STIs within 12 months prior to initial doxy PEP Rx, no. (%)** |  |  | 0.805 |
| 0 | 12 (44.4%) | 25 (39.7%) |  |
| 1 | 9 (33.3%) | 19 (30.2%) |  |
| >1 | 6 (22.2%) | 19 (30.2%) |  |
| **Provider ordering Rx, no. (%)** |  |  | 0.228 |
| Pharmacist | 18 (66.7%) | 29 (46%) |  |
| APP/ID Attending | 6 (22.2%) | 24 (38.1%) |  |
| ID Fellow | 3 (11.1%) | 10 (15.9%) |  |
| **Proportion of days covered (%), mean + SD** | 42 + 33.4% | 43 + 33.2% | 0.921 |
| **Doses prescribed on initial doxy PEP Rx, mean + SD** | 7.5 + 4.8 | 12 + 9.5 | **0.004** |
|  | **No STI while prescribed doxy PEP**  **(N=77)** | **STI while prescribed doxy PEP**  **(N=13)** |  |
| **Age (years), mean + SD** | 39.6 + 10.5 | 32.2 + 8.1 | **0.022** |
| **Race, no. (%)** |  |  | 0.408 |
| White | 55 (71.4%) | 7 (53.8%) |  |
| Black | 11 (14.3%) | 3 (23.1%) |  |
| Other | 11 (14.3%) | 3 (23.1%) |  |
| **Ethnicity, no. (%)** |  |  | 1 |
| Non-Hispanic | 61 (79.2%) | 11 (84.6%) |  |
| Hispanic | 16 (20.8%) | 2 (15.4%) |  |
| **Insurance coverage, no. (%)** |  |  | 0.229 |
| Commercial/Private | 51 (66.%) | 6 (46.2%) |  |
| Government-funded/ADAP | 20 (26%) | 6 (46.2%) |  |
| Uninsured | 6 (7.8%) | 1 (7.7%) |  |
| **Reason for care at SCC, no. (%)** |  |  | **0.038** |
| PrEP | 43 (55.8%) | 3 (23.1%) |  |
| HIV | 34 (44.2%) | 10 (76.9%) |  |
| **Number of STIs within 12 months prior to initial doxy PEP Rx, no. (%)** |  |  | **0.019** |
| 0 | 36 (46.8%) | 1 (7.7%) |  |
| 1 | 22 (28.6%) | 6 (46.2%) |  |
| >1 | 19 (24.7%) | 6 (46.2%) |  |
| **Proportion of days covered (%), mean + SD** | 42 + 33.3 | 48 + 32.4 | 0.487 |
| **Doses prescribed on initial doxy PEP Rx, mean + SD** | 10 + 8.2 | 15 + 10 | 0.062 |

Abbreviations: ADAP = AIDS Drug Assistance Program; APP = advanced practice provider; doxy PEP = doxycycline post-exposure prophylaxis; HIV = human immunodeficiency virus; ID = infectious diseases; no. = number; PrEP = pre-exposure prophylaxis; Rx = prescription; SCC = University of Nebraska Medical Center Specialty Care Clinic; SD = standard deviation; STI = sexually transmitted infection
